# Comparative Outcomes of Early Surgical Intervention Versus Nonoperative Management in Adhesive Small Bowel Obstruction

**DOI:** 10.7759/cureus.100991

**Published:** 2026-01-07

**Authors:** Muhammad Sanwal Abrar, Arslan Shahid, Abdullah Madni, Muhammad Nouman, Muhammed Mustafa, Hafiz Muhammad Usman, Ammar Yasir, Fatima Zafar, Tahawwur Zubair, Muhammad Arshad Abbas

**Affiliations:** 1 Surgery, Shahida Islam Teaching Hospital, Lodhran, PAK; 2 General Surgery, Dost Muhammed Awan Hospital, Basti Malook, PAK; 3 Medicine, Shahida Islam Medical College, Lodhran, PAK; 4 Emergency Medicine, Reliance Hospital Civic Center, Islamabad, PAK; 5 Biochemistry, Shaida Islam Medical College, Lodhran, PAK; 6 Care of the Elderly, Warrington and Halton Hospitals NHS Trust, England, GBR; 7 General Surgery, Shahida Islam Teaching Hospital, Lodhran, PAK

**Keywords:** adhesive small bowel obstruction, bowel disease, comparison, early surgical intervention, non-operative management

## Abstract

Background: Adhesive small bowel obstruction (ASBO) is a common cause of intestinal obstruction, often managed either through nonoperative strategies or early surgical intervention.

Objective: To compare the outcomes of nonoperative management versus early surgery in patients presenting with ASBO.

Methodology: This comparative observational study was conducted at Shahida Islam Teaching Hospital, Lodhran, from February 2024 to August 2024. A total of 201 patients with clinically and radiologically confirmed ASBO were included through non-probability consecutive sampling. Patients were divided into two groups: Group A received nonoperative management, while Group B underwent early surgical intervention within 24 hours of admission.

Results: Among 201 patients, 120 (59.7%) were managed nonoperatively and 81 (40.3%) underwent early surgery. Clinical resolution without surgery was achieved in 104/120 (86.7%) patients in the nonoperative group. Complications occurred in 6/120 (5.0%) patients in the nonoperative group versus 23/81 (28.4%) in the surgical group (*P* = 0.02). The mean hospital stay was significantly shorter in the nonoperative group (4.6 ± 1.8 days) compared to the early surgery group (8.1 ± 2.5 days, *P* < 0.001). However, recurrence within six months was significantly higher in the nonoperative group (26/120, 21.7%) compared to the early surgery group (7/81, 8.6%) (*P* = 0.01).

Conclusions: Nonoperative management remains effective and safe for uncomplicated ASBO, offering shorter hospitalization and fewer immediate complications. Although early surgical intervention was associated with a higher complication rate, this finding likely reflects the inclusion of patients with more advanced disease at presentation rather than surgical risk alone. Early operative management remains essential for complicated or non-resolving cases to prevent ischemic injury and recurrence.

## Introduction

Adhesive small bowel obstruction (ASBO) represents one of the most frequent indications for emergency surgical consultation and inpatient care globally, particularly in developed healthcare settings where prior surgical history is common [1,2]. ASBO occurs when fibrous bands or adhesions form between loops of bowel or between the bowel and abdominal wall following surgical interventions, infections, or inflammatory processes. These adhesions disrupt the normal passage of intestinal contents, leading to varying degrees of obstruction. According to recent estimates, SBO is a common surgical emergency, contributing to 15% to 20% of hospital admissions for acute abdominal pain and accounting for approximately 80% of all bowel obstructions [3]. The standard management of ASBO is centered around two main approaches: nonoperative management (NOM) and early surgical intervention. NOM is widely accepted as the initial treatment modality in hemodynamically stable patients without signs of peritonitis, strangulation, or ischemia [4,5]. The NOM protocol typically includes bowel rest, nasogastric decompression, intravenous fluid resuscitation, correction of electrolyte imbalances, and close clinical monitoring. The rationale behind favoring NOM lies in its ability to avoid surgical risks, reduce healthcare costs, and minimize patient morbidity associated with repeat laparotomies and potential iatrogenic bowel injury. Various studies have reported that up to 70%-80% of uncomplicated ASBO cases resolve successfully without surgery within 48-72 hours of conservative treatment [6].

However, NOM is not without limitations. One major concern is the risk of delayed recognition of bowel ischemia or necrosis, which may occur silently in the early stages and may only become clinically apparent once irreversible damage has occurred. Prolonged conservative management in such cases may result in increased morbidity, the need for more extensive surgical resections, and even mortality [7]. Additionally, patients with ASBO managed nonoperatively may experience higher recurrence rates, necessitating future hospital admissions and interventions, which have implications for both patient quality of life and healthcare system burden [8]. Early surgical intervention, on the other hand, aims to promptly relieve obstruction and address any underlying complications such as bowel strangulation or perforation. Surgery typically involves laparotomy or laparoscopic adhesiolysis, removal of adhesive bands causing the obstruction [9]. Advocates for early surgery argue that timely operative management can reduce hospital length of stay, prevent recurrence, and mitigate risks of bowel ischemia by addressing the pathology before irreversible damage occurs. However, surgery carries its own set of risks, including postoperative infections, iatrogenic bowel injury, incisional hernias, prolonged recovery time, and increased financial costs [10,11]. The debate between nonoperative and early operative management of ASBO continues in surgical literature and clinical practice, with no universal consensus. Some guidelines recommend a trial of NOM for 48-72 hours in uncomplicated cases, while others suggest early surgery based on patient-specific risk factors such as age, comorbidities, and radiological findings. Recent advances in imaging, such as contrast-enhanced computed tomography (CT), have improved the ability to stratify patients according to risk, aiding in decision-making regarding the timing of intervention. 

Therefore, the main objective of the study is to compare the short-term outcomes of NOM versus early surgery in patients presenting with ASBO. 

## Materials and methods

Methodology

This was a comparative observational study conducted at Shahida Islam Teaching Hospital, Lodhran, from February 2024 to August 2024. A total of 201 patients diagnosed with adhesive small bowel obstruction were included in the study. Non-probability consecutive sampling was used to recruit all eligible patients. Patients aged 18 years or older of either gender with clinically and radiologically confirmed adhesive small bowel obstruction and a documented history of prior abdominal or pelvic surgery were included in the study. Patients presenting with peritonitis or bowel ischemia requiring surgery were included in the surgical arm, as these conditions represented the actual clinical indications for operative intervention. Patients with bowel obstruction due to malignancy, hernia, volvulus, or inflammatory bowel disease were excluded. Those with advanced systemic illnesses such as uncontrolled diabetes, liver or renal failure, or severe cardiac disease were also omitted. 

Data collection

Data were collected using a structured proforma designed specifically for the study. Enrolled patients were divided into two groups based on the initial management strategy employed by the attending surgical team. Group A consisted of patients managed nonoperatively, which involved bowel rest, nasogastric tube decompression, intravenous fluid administration, electrolyte correction, and close monitoring for clinical improvement or deterioration. Group B comprised patients who underwent early surgical intervention, defined as operative management performed within 24 hours of hospital admission. The decision regarding group allocation was based on clinical judgment, radiological assessment, and institutional management protocols. Variables recorded included patient demographics such as age and gender, presenting symptoms, radiological findings, type of management received, duration of hospital stay, postoperative complications such as wound infection or bowel injury, recurrence of adhesive small bowel obstruction within six months of discharge, and in-hospital mortality. Recurrence of adhesive small bowel obstruction within six months of discharge was assessed through outpatient clinic follow-up visits and review of hospital readmission records. 

Data analysis

All collected data were entered into the SPSS software version 26 (IBM Corp., Armonk, NY) for statistical analysis. Descriptive statistics were applied to summarize baseline characteristics, with continuous variables expressed as mean ± standard deviation and categorical variables presented as frequencies and percentages. The independent samples t-test was applied to compare continuous variables between the nonoperative and early surgery groups, and the chi-square or Fisher’s exact test was used for categorical variables as appropriate. A *P*-value less than 0.05 was considered statistically significant for all tests.

## Results

Data were collected from 201 participants, with a mean age of 52.4 ± 14.7 years in the overall cohort, 51.8 ± 13.9 years in the NOM group, and 53.2 ± 15.5 years in the early surgery group. Out of the total, 124 patients (61.7%) were male, and 77 (38.3%) were female. In the nonoperative group, there were 74 males (61.7%) and 46 females (38.3%), while in the early surgery group, there were 50 males (61.7%) and 31 females (38.3%). Regarding previous abdominal surgeries, appendectomy was the most common, reported in 86 (42.8%): 51 (42.5%) patients in the nonoperative group and 35 (43.2%) in the surgical group. Cesarean section was performed in 57 patients (28.4%), including 36 (30.0%) in the nonoperative and 21 (25.9%) in the surgical group, while colorectal surgery was reported in 36 patients (17.9%), with 21 (17.5%) in the nonoperative and 15 (18.5%) in the early surgery group (Table 1).

**Table 1:** Baseline demographic and clinical characteristics of study participants. SD, standard deviation

Characteristics	Total (*n* = 201)	Nonoperative management (*n* = 120)	Early surgery (*n* = 81)
Age (years), mean ± SD	52.4 ± 14.7	51.8 ± 13.9	53.2 ± 15.5
Gender, *n* (%)			
Male	124 (61.7%)	74 (61.7%)	50 (61.7%)
Female	77 (38.3%)	46 (38.3%)	31 (38.3%)
Previous abdominal surgery, *n* (%)			
Appendectomy	86 (42.8%)	51 (42.5%)	35 (43.2%)
Cesarean section	57 (28.4%)	36 (30.0%)	21 (25.9%)
Colorectal surgery	36 (17.9%)	21 (17.5%)	15 (18.5%)
Others	22 (10.9%)	12 (10.0%)	10 (12.3%)

Clinical resolution without the need for surgical intervention was achieved in 104 out of 120 patients (86.7%) managed nonoperatively, while 16 patients (13.3%) required delayed surgery due to failure of conservative management. The mean hospital stay was significantly longer among patients in the early surgery group (8.1 ± 2.5 days) compared to those managed nonoperatively (4.6 ± 1.8 days), with a *P*-value < 0.001 indicating statistical significance (Table 2).

**Table 2:** Management outcomes and hospital stay. *P*-value <0.05 is considered statistically significant. An asterisk (*) denotes statistical significance.

Outcome	Nonoperative (*n* = 120)	Early surgery (*n* = 81)	Test statistics	*P*-value
Clinical resolution, *n* (%)	104 (86.7%)	-	-	-
Required delayed surgery, *n* (%)	16 (13.3%)	-	-	-
Mean hospital stay (days)	4.6 ± 1.8	8.1 ± 2.5	*T* = 10.42	<0.001*

Postoperative and in-hospital complications were notably higher in the early surgery group, where 23 patients (28.4%) experienced at least one complication compared to 6 patients (5.0%) in the nonoperative group (*P* < 0.001). The most frequent surgical complications included wound infection in 10 patients (12.3%), postoperative ileus in 6 (7.4%), and iatrogenic bowel injury in 3 (3.7%). In contrast, the nonoperative group mainly experienced minor or medical complications, such as aspiration pneumonia (1, 0.8%), acute kidney injury (2, 1.7%), and electrolyte imbalance (1, 0.8%) (Table 3).

**Table 3:** Complications and mortality. *P*-value <0.05 is considered statistically significant. *P < 0.05 (significant). **P < 0.001 (highly significant).

Complication type	Nonoperative (*n* = 120)	Early surgery (*n* = 81)	Test statistic (Fisher’s exact)	*P*-value
Major surgical complications				
Wound infection	0 (0.0%)	10 (12.3%)	13.26	<0.001*
Postoperative ileus	0 (0.0%)	6 (7.4%)	7.18	0.007*
Iatrogenic bowel injury	0 (0.0%)	3 (3.7%)	3.96	0.041*
Major medical complications				
Aspiration pneumonia	1 (0.8%)	-	-	-
Acute kidney injury	2 (1.7%)	1 (1.2%)	0.00	0.95
Electrolyte imbalance	1 (0.8%)	-	-	-
Minor complications				
Nausea/vomiting	1 (0.8%)	2 (2.5%)	0.01	0.94
Low-grade fever	1 (0.8%)	1 (1.2%)	0.00	0.95
Total patients with ≥ 1 complication	6 (5.0%)	23 (28.4%)	19.88	<0.001**

The most common indication was signs of peritonitis (22, 27.2%), followed by bowel ischemia on imaging (18, 22.2%) and failure of conservative management (16, 19.8%). Other indications included persistent vomiting or dehydration (14, 17.3%) and severe abdominal distension (11, 13.5%) (Table 4).

**Table 4:** Indications for early surgery. CT, computed tomography

Indication for surgery	Frequency (*n*)	Percentage (%)
Signs of peritonitis	22	27.2
Bowel ischemia (suspected/confirmed)	18	22.2
Complete bowel obstruction (no gas/stool passage)	21	25.9
Recurrent obstruction with severe distension	12	14.8
Closed loop or strangulated obstruction (CT confirmed)	8	9.9
Total	81	100.0

Dilated small bowel loops were observed in 115 (95.8%) patients in the nonoperative group and 74 (91.4%) patients in the early surgery group (χ² = 1.79, *P* = 0.18). In comparison, multiple air-fluid levels were noted in over 93% of both cohorts (χ² = 0.01, *P* = 0.91). A transition point was identified in 92 (76.7%) nonoperative cases and 69 (85.2%) early surgical cases (χ² = 2.17, *P* = 0.14), indicating similar radiologic obstruction patterns. However, free peritoneal fluid was significantly more common in the surgical group (37, 45.7%) than in the nonoperative group (38, 31.7%) (χ² = 4.14, *P* = 0.04), suggesting a greater likelihood of complicated obstruction. Likewise, CT evidence of closed-loop or strangulated obstruction was significantly more common in the surgical cohort (17.3% vs. 6.7%; χ² = 5.46, *P* = 0.02) (Table 5).

**Table 5:** Radiologic findings in patients with adhesive small bowel obstruction (ASBO). *P*-value <0.05 is considered statistically significant. An asterisk (*) denotes significance.

Radiologic findings	Nonoperative (*n* = 120)	Early surgery (*n* = 81)	Total (*n* = 201)	Χ²	*P*-value
Dilated small bowel loops	115 (95.8%)	74 (91.4%)	189 (94.0%)	1.79	0.18
Multiple air-fluid levels	112 (93.3%)	76 (93.8%)	188 (93.5%)	0.01	0.91
Transition point identified	92 (76.7%)	69 (85.2%)	161 (80.1%)	2.17	0.14
Free peritoneal fluid	38 (31.7%)	37 (45.7%)	75 (37.3%)	4.14	0.04*
Closed loop/strangulation on CT	8 (6.7%)	14 (17.3%)	22 (10.9%)	5.46	0.02*
No bowel dilatation (atypical presentation)	5 (4.2%)	7 (8.6%)	12 (6.0%)	1.45	0.23

## Discussion

This study was conducted to compare the outcomes of NOM versus early surgical intervention in patients presenting with ASBO. A total of 201 patients were included, divided into NOM and early surgery groups, allowing assessment of the relative benefits and risks of both approaches. Our findings revealed that NOM was successful in 120 (86.7%) patients, aligning with previous research where nonoperative success rates typically range from 70% to 85% in uncomplicated cases with ASBO. The mean hospital stay was significantly longer in the early surgery group compared to the nonoperative group, aligning with the international literature, which indicates that operative intervention is associated with an increased length of stay due to postoperative recovery and complication management. These results reinforce the value of a trial of NOM in stable ASBO patients without signs of peritonitis or ischemia [12,13].

However, this benefit must be weighed against the notably higher recurrence rate observed in the nonoperative group. In our study, 6.5% of conservatively managed patients experienced recurrence within six months compared to only 8.6% in the early surgery group, a statistically significant difference. Similar patterns have been documented in previous research, which suggests that while nonoperative treatment avoids immediate surgical risks, it may predispose patients to recurrent admissions and long-term morbidity [14,15]. This finding emphasizes the importance of individualized patient selection for conservative treatment, considering factors such as previous obstruction episodes and patient comorbidities. Complication rates were significantly higher in the early surgery group (57, 28.4%) compared to the nonoperative group (6, 5%), mainly due to surgical site infections, postoperative ileus, and iatrogenic bowel injuries. These observations align with existing literature that identifies adhesiolysis as a procedure carrying considerable morbidity risk. Despite this, early surgery resulted in a lower recurrence rate and effectively addressed cases with imaging-confirmed bowel ischemia or failed conservative management. This duality echoes the conclusions from other studies that advocate for early operative intervention in selected high-risk cases while reserving conservative treatment for uncomplicated presentations.

The in-hospital mortality rate in our study was relatively low at 3%, with no statistically significant difference between the two groups. This finding is comparable to previous research where overall mortality in patients with ASBO remains below 5% when managed according to established protocols [16,17]. It is noteworthy that all deaths in the early surgery group were related to complications arising from delayed intervention, underscoring the importance of timely clinical decision-making. Additionally, our data show that radiological findings such as a visible transition point and signs of bowel ischemia were significantly associated with early surgery, reflecting the current clinical practice of relying on imaging to guide management strategy. This highlights the pivotal role of advanced imaging in stratifying patients with ASBO into appropriate treatment pathways. The study was limited by its observational design and single-center setting, which may affect the generalizability of the results. Group allocation was based on clinician discretion rather than randomization, introducing potential selection bias. Furthermore, long-term follow-up beyond six months was not conducted, so the recurrence rate reported may underestimate late presentations.

## Conclusions

It is concluded that NOM was successful in the majority of patients with ASBO, whereas early surgical intervention was required in cases presenting with peritonitis, ischemia, or failure of conservative therapy. Although early surgery was associated with longer hospital stays and higher complication rates, this relationship is likely influenced by confounding by indication, as patients in the surgical group had more severe disease at presentation. Therefore, the poorer outcomes observed among surgically treated patients reflect the underlying disease severity rather than the procedure itself. Careful patient selection and prompt recognition of clinical or radiologic signs of complicated obstruction are essential to optimizing outcomes.
